# Improved Measurement of Blood Pressure by Extraction of Characteristic Features from the Cuff Oscillometric Waveform

**DOI:** 10.3390/s150614142

**Published:** 2015-06-16

**Authors:** Pooi Khoon Lim, Siew-Cheok Ng, Wissam A. Jassim, Stephen J. Redmond, Mohammad Zilany, Alberto Avolio, Einly Lim, Maw Pin Tan, Nigel H. Lovell

**Affiliations:** 1 Department of Biomedical Engineering, Faculty of Engineering, University of Malaya, 50603 Kuala Lumpur, Malaysia; E-Mails: veronicalimpooikhoon@gmail.com (P.K.L); siewcng@um.edu.my (S.-C.N.); wissam@um.edu.my (W.A.J.); zilany@um.edu.my (M.Z.);; 2 Graduate School of Biomedical Engineering, UNSW Australia, Sydney, NSW 2052, Australia; E-Mails: s.redmond@unsw.edu.au (S.J.R.); N.Lovell@unsw.edu.au (N.H.L.); 3 Department of Biomedical Sciences, Faculty of Medicine and Health Sciences, Macquarie University, Sydney, NSW 2109, Australia; E-Mail: alberto.avolio@mq.edu.au; 4 Department of Medicine, Faculty of Medicine, University of Malaya, 50603 Kuala Lumpur, Malaysia; E-Mail: mptan@ummc.edu.my

**Keywords:** oscillometric blood pressure estimation, multiple linear regression, support vector regression

## Abstract

We present a novel approach to improve the estimation of systolic (SBP) and diastolic blood pressure (DBP) from oscillometric waveform data using variable characteristic ratios between SBP and DBP with mean arterial pressure (MAP). This was verified in 25 healthy subjects, aged 28 ± 5 years. The multiple linear regression (MLR) and support vector regression (SVR) models were used to examine the relationship between the SBP and the DBP ratio with ten features extracted from the oscillometric waveform envelope (OWE). An automatic algorithm based on relative changes in the cuff pressure and neighbouring oscillometric pulses was proposed to remove outlier points caused by movement artifacts. Substantial reduction in the mean and standard deviation of the blood pressure estimation errors were obtained upon artifact removal. Using the sequential forward floating selection (SFFS) approach, we were able to achieve a significant reduction in the mean and standard deviation of differences between the estimated SBP values and the reference scoring (MLR: mean ± SD = −0.3 ± 5.8 mmHg; SVR and −0.6 ± 5.4 mmHg) with only two features, *i.e.*, Ratio_2_ and Area_3_, as compared to the conventional maximum amplitude algorithm (MAA) method (mean ± SD = −1.6 ± 8.6 mmHg). Comparing the performance of both MLR and SVR models, our results showed that the MLR model was able to achieve comparable performance to that of the SVR model despite its simplicity.

## 1. Introduction

Blood pressure, commonly expressed in terms of systolic (maximum) and diastolic (minimum) pressures, offers important insights into cardiovascular health. High blood pressure (hypertension), which may lead to stroke and heart failure, has been rated as one of the most important causes of premature death by the World Health Organization [1]. On the other hand, excessively low blood pressure (hypotension) may indicate underlying diseases such as heart failure and adrenal insufficiency [2]. Thus, noninvasive measurement of blood pressure using either auscultatory or oscillometric methods are routinely performed [3].

The auscultatory measurement using the mercury sphygnomanometer, which estimates systolic (SBP) and diastolic blood pressure (DBP) using the Korotkoff sounds, has been widely accepted as the gold standard [4,5]. Despite its highly accurate and reliable blood pressure measurement, the auscultatory method is not commonly used for automated estimation of blood pressure [6] as it requires a trained professional. Furthermore the mercury sphygmomanometer is gradually being withdrawn from clinical use. The oscillometric method, on the other hand, has become increasingly popular in automated blood pressure measurement devices [6]. An electronic pressure sensor is used to observe the pressure oscillation in the cuff during its gradual deflation from above SBP to below DBP. The oscillation amplitude increases to its maximum value when the cuff pressure reaches the mean arterial pressure (MAP), and then gradually decreases with further deflation of the cuff pressure [7]. The upper envelope of the oscillometric waveform is known as the oscillometric waveform envelope (OWE).

Conventionally, the SBP and DBP values are estimated from the OWE using the maximum amplitude algorithm (MAA) either with the slope-based or height-based method [8]. The main drawback of the slope-based method is that it defines SBP and DBP as the cuff pressure corresponding to the maximum slope of increasing and decreasing amplitude of the OWE, which are not well defined and thus constraints have to be applied to estimate SBP with an acceptable accuracy [8]. On the other hand, height-based method linearly relates the SBP and the DBP to the mean blood pressure using fixed empirically derived height (or characteristic) ratios [9].

The estimation of SBP and DBP using this experimentally-derived, quasi-empirical characteristic ratio is prone to error as it is subject to significant continuous variability over time [10,11,12]. Furthermore, the characteristic ratio has been reported to be sensitive to changes in physiological conditions, in particular the degree of arterial stiffness [12,13,14]. For instance, the fixed-ratio method overestimates SBP but underestimates DBP in individuals with stiffening of the brachial artery [12]. Despite these findings, very limited studies have assessed alternative methods to improve the accuracy of SBP and DBP measurements. Feature-based Gaussian mixture regression approach [15] as well as neural network [16], Bayesian model [7], and a statistical learning technique based on logistic regression [17] were among the alternative methods. Five features, such as MAP, maximum amplitude, length of the maximum amplitude’s position, length of OWE and asymmetry ratio of the OWE were used to estimate SBP and DBP using the Gaussian mixture regression model [15].

In the present study, we evaluated the performance of ten features from the OWE, which included previously used features in addition to newly proposed features, in describing the systolic (SBPR) and diastolic blood pressure ratio (DBPR). Furthermore, we attempted to minimize the usage of multiple features by applying the sequential forward floating selection (SFFS) method and to identify the combination of features that result in the best performance. Two different models, using multiple linear regression (MLR) and support vector regression (SVR) methods were used to estimate SBP and DBP. Carefully designed experiments were performed to obtain noise-free signals and signals containing noise induced by movement so as to evaluate the robustness of the algorithm to motion artifact, commonly occurring in an unsupervised environment. A pre-processing step was carried out to detect and eliminate data points corrupted by movement artifact.

The paper is organized as follows: the methodology for this paper is explained in Section 2; the effect of noise detection (outlier removal) and performance of blood pressure estimation using conventional MAA, MLR and SVR models is presented in Section 3; result are discussed in Section 4 followed by conclusion in Section 5.

## 2. Experimental Section

**Figure 1:**
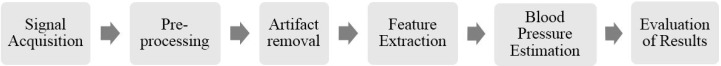
Block diagram of sequence of events in blood pressure estimation.

Figure 1 shows the sequence of events in blood pressure estimation.

### 2.1. Signal Acquisition

**Figure 2:**
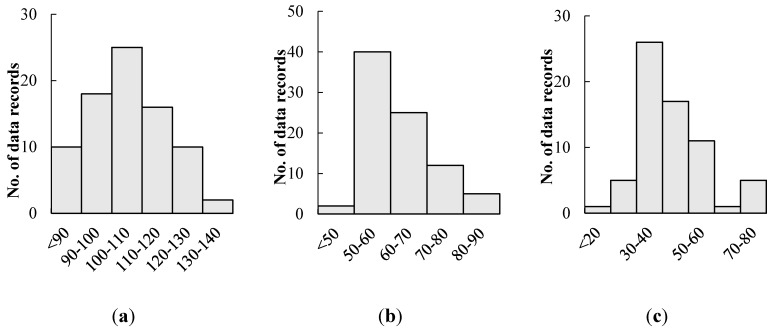
Distribution of (**a**) Systolic blood pressure (SBP); (**b**) Diastolic blood pressure (DBP); (**c**) Pulse pressure (PP).

The experimental data were obtained from 25 healthy subjects aged 28 ± 5 years (16 females). Four sets of measurements (two from each arm), which contain simultaneous ECG, cuff pressure and Korotkoff sound were acquired from each volunteer, resulting in a total of 100 measurements. Our data were acquired using an automated blood pressure measurement system with a cuff pressure recorder, a stethoscope with a built-in microphone to capture the auscultatory waveform, together with an ECG recorder. All the signals were acquired simultaneously using a data acquisition system with a sampling rate of 1 kHz. To acquire the oscillometric pulse, the cuff pressure was first increased to approximately 180 mmHg, followed by deflation of the cuff pressure using a release valve, which reduced the pressure to approximately 40 mmHg in a linear fashion and with a rate of 2–3 mmHg/s. To investigate the robustness of the BP estimation algorithm, one of the two measurements on each arm was intentionally contaminated with movement artifact during cuff deflation. The movements were selected from the following options: (1) gently lift the ipsilateral arm, then return to a resting position; (2) spontaneously move the ipsilateral arm right and left; (3) bend the ipsilateral arm and then return to a resting position; (4) tap the stethoscope bell three times with the contralateral hand; (5) squeeze and release the ipsilateral fingers; (6) lift and replace a book with the ipsilateral hand; (7) spontaneously shake the ipsilateral arm for a few seconds; and (8) suddenly remove the cuff. The recorded Korotkoff sound was used by two clinical experts as the basis for estimating the reference SBP and DBP as a reference system (RS). Out of the 100 signals, only 81 SBP and 84 DBP were available for this study due to a lack of reference reading in the remaining samples, in which the experts were unable to identify the SBP and DBP accurately due to the presence of a large amount of noise in the Korotkoff sound. Figure 2 shows the distribution of SBP, DBP and pulse pressure (PP) in the collected data. A more detailed description of the experimental protocol as well as equipment configuration are provided in [18].

### 2.2. Pre-Processing

**Figure 3:**
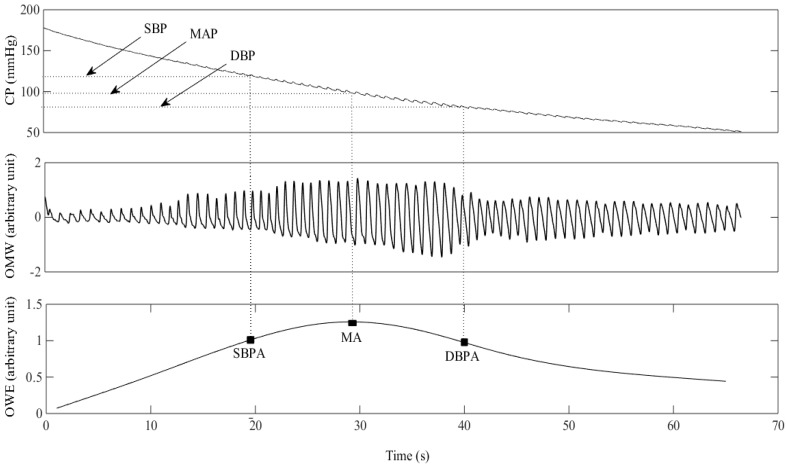
An example of the deflating cuff pressure (CP) waveform, pulsatile oscillometric waveform (OMW), and oscillometric waveform envelope (OWE). MA: Amplitude of the OWE corresponding to the location of the mean arterial pressure (MAP); SBPA: Amplitude of the OWE corresponding to the location of the systolic blood pressure (SBP); DBPA: Amplitude of the OWE corresponding to the location of the diastolic blood pressure (DBP).

The cuff pressure signal was detrended using a first-order band-pass Butterworth filter of 0.5–5 Hz, chosen based on the assumption of a maximum heart rate of 300 beats per minute [18] to transform the signal morphology into a pulsatile oscillometric waveform. A forward-backward filter was used to achieve a zero-phase response. Since the ECG signals were not affected by the movement of the subjects, the intervals between two consecutive R-peaks in the ECG waveforms were used to determine each cardiac cycle. Figure 3 illustrates an example of the cuff pressure signal, pulsatile oscillometric waveform and its corresponding OWE. The reference systolic blood pressure ratio (SBPR) and diastolic blood pressure ratio (DBPR) were extracted from the OWE and were defined as follows:

(1) $$SBPR=\frac{SBPA}{MA}$$

(2) $$DBPR=\frac{DBPA}{MA}$$

where MA represents the maximum amplitude of the OWE corresponding to the location of the MAP, while SBPA and DBPA indicate the amplitudes of the OWE corresponding to the location of the SBP and the DBP respectively.

**Figure 4:**
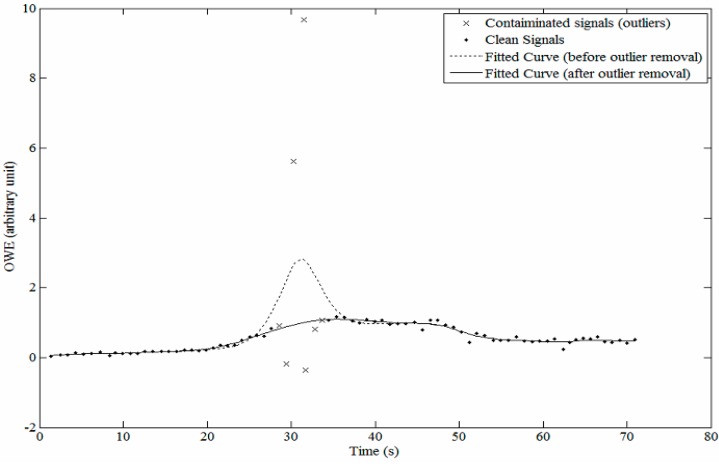
Cubic spline curve fitted to the oscillometric waveform envelope (OWE) before and after removal of outlier pulses.

### 2.3. Detection and Removal of Outlier Points

A cubic spline curve was used to fit the OWE [7]. In order to increase the accuracy of the SBP and DBP estimation, data points contaminated with motion artifact were treated as outliers and removed during the OWE curve fitting process. First, an automatic algorithm was used to detect these outlier points based on the suddenly increase of cuff pressure during deflation and the oscillometric pulses relative to their respective neighbour pulses. The peak, peak-to-peak, peak-to-bottom and bottom points of every oscillometric pulses were investigated. To be considered as clean data pulses, the absolute variations of the heights should not be more than 0.4 and the height of each of these points should lie within ±50% of their respective neighbour pulses based on modification of [19]. Besides that, a suddenly increasing pressure during cuff deflation will also be considered as artifact. Figure 4 illustrates the effect of outlier removal on the fitted curve for the OWE.

### 2.4. Feature Extraction

**Figure 5:**
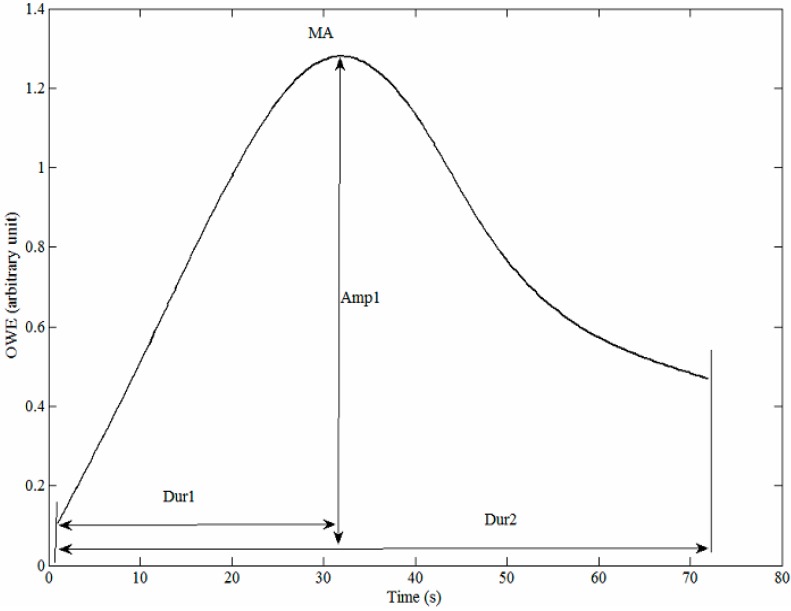
Description of features extracted from the OWE.

In the present study, a total of 10 features were extracted from the OWE, as illustrated in Figure 5 and defined in Table 1, in which six have been used in a previous study [15], whereas the remaining features were newly proposed in this study. These features can be classified into five different classes: (I) Amplitude; (II) Duration; (III) Area; (IV) Ratio; and (V) MAP estimated using the MAA approach.

**Table 1:** Description of features extracted from the OWE. The * symbol in the references column refers to features proposed in this study Description/ Equation

Feature	Description/Equation	References
Amp_1_	Maximum Amplitude of OWE	[15]
Dur_1_	Duration for maximum amplitude (MA) to occur	[15]
Dur_2_	Duration of OWE	[15]
Area_1_	Area under OWE	[15]
Area_2_	Area under OWE before the MA’s position	*
Area_3_	Area under OWE after the MA’s position	*
Ratio_1_	Duration for maximum amplitude to occur/Duration of OWE	[15]
Ratio_2_	Area under OWE before the MA’s position/Area under OWE	*
Ratio_3_	Area under OWE after the MA’s position/Area under the OWE	*
MAP	MAP estimated using the MAA algorithm	[15]

Features from the amplitude class have been previously proposed by Lee *et al.* [15]. Amp_1_ was motivated by the theoretical analysis findings by Baker which demonstrates the dependence of MAA estimates on the arterial mechanical properties, blood pressure pulse shape and blood pulse pressure [9]. The second class of features was derived based on duration. Dur_1_ and Dur_2_ were motivated by their other study [20], which demonstrated an improvement in the SBP and DBP estimates using the new relationships between the mean cuff pressure and the pseudoenvelopes that relate the duration of the MA’s position and OWE. The third class of features was derived based on area measurements. The area under OWE (Area_1_) was proposed by Lee *et al.* [15] based on Baker *et al.*’s [9] findings, and this led us to propose two other relevant features, *i.e.*, the area before (Area_2_) and after (Area_3_) the MA’s position. The third class of features were derived based on the morphology of the OWE, which demonstrated the dependence of the SBP and DBP estimates on the shape of the OWE [9]. The ratio between the duration of the MA’s position to duration of OWE (Ratio_1_) was proposed by Lee *et al.* [15], while two other features were newly proposed in the present study based on the modification of Ratio_1_. Instead of relying on the position of the MA, Ratio_2_ and Ratio_3_ also took into consideration the height of the OWE curve by relating the area under the OWE before and after the MA’s position to the area under the OWE. The last feature, *i.e.*, MAP, has also been previously proposed by Lee *et al.* [15], based on Moraes’ findings [21] which indicated a close correlation between SBPR and DBPR with the MAP values.

### 2.5. Blood Pressure Estimation Models

Three different blood pressure estimation models were evaluated in the present study, including the conventional Maximum Amplitude Algorithm (MAA) method based on fixed characteristic ratios, and two newly proposed models were obtained using multiple linear regression (MLR) and support vector regression (SVR).

#### 2.5.1. Maximum Amplitude Algorithm (MAA)

The conventional MAA method based on a fixed characteristic ratio were used to determine SBP and DBP. The fixed SBPR and DBPR were obtained as averages of the SBPR and DBPR derived from our RS.

#### 2.5.2. Multiple Linear Regression (MLR) Model

MLR was used to model the relationship between the SBPR and DBPR with the features extracted from the OWE, and is defined as follows:

(3) $$y=\;b_{0}+b_{1}x_{1}+b_{2}x_{2}+\ldots +b_{n}x_{n}+\text{ε}$$

where *y* denotes either SBPR or DBPR, *x* denotes the input features, *b* denotes the multiple regression coefficients, while ε is a sequence of unknown errors. Depending on the number of measurements, denoted by *p*, a matrix form containing information from each measurement will be defined as below [22]:

(4) Y=Xβ+E

(5) $$\text{Y}=\left(\begin{array}{c} \begin{array}{c} {\text{y}}_{1}\\ {\text{y}}_{2} \end{array}\\ \begin{array}{c} .\\ .\\ .\\ {\text{y}}_{p} \end{array} \end{array}\right)\text{ X}=\left(\begin{array}{c} \begin{array}{c} {\text{x}}_{11}\;X{\text{x}}_{1\text{p}}\\ {\text{x}}_{21}\;X{\text{x}}_{2\text{p}} \end{array}\\ \begin{array}{c} \;.\;.\;\\ \;.\;.\;\\ \;.\;.\;\\ {\text{x}}_{\text{n}1}\;.\;{\text{x}}_{\text{np}} \end{array} \end{array}\right)\text{ β}=\left(\begin{array}{c} \begin{array}{c} {\text{b}}_{1}\\ {\text{b}}_{2} \end{array}\\ \begin{array}{c} .\\ .\\ .\\ {\text{b}}_{p} \end{array} \end{array}\right)\text{ E}=\left(\begin{array}{c} \begin{array}{c} {\text{ε}}_{1}\\ {\text{ε}}_{2} \end{array}\\ \begin{array}{c} .\\ .\\ .\\ {\text{ε}}_{p} \end{array} \end{array}\right)$$

The multiple regression coefficients, β, can then be obtained based on the minimum sum of squared errors by solving:

(6) $$y=\;b_{0}+b_{1}x_{1}+b_{2}x_{2}+\ldots +b_{n}x_{n}+\text{ε}$$

(7) $$\text{β}={\left({\text{X}}^{T}\text{X}\right)}^{-1}{\text{X}}^{T}\text{Y}$$

#### 2.5.3. Linear v-Support Vector Regression (ν-SVR) Model

Consider a set of training points, {(*x*_1_, *y*_1_), …, (*x_l_*, *y_l_*)}, where *x*_i_ ∈ $R^{n}$ is a feature vector while y_i_ ∈ $R^{1}$ is the target output. The ν- SVR model searches for the best approximation of the actual output y_i_ (*i.e.*, SBPR and DBPR in the present study) based on the input features, *x_i_*, with an acceptable error tolerance of ɛ. Let *x_i_* be mapped into a feature space by a nonlinear function φ(x); the decision function becomes:

(8) y=f(w,b)=w.φ(x)+ b

where *w* and *b* are parameters vectors of the SVR model. The parameter *w* vector determines the flatness of the approximation function, with lower *w* values giving smoother and less complicated approximation function [23,24]. The mapping function φ(x) transforms the data into a higher dimensional feature space to make it possible to perform the linear separation. Parameter ν ∈(0,1] is used to control the number of support vectors and training errors. The regression problem was formulated as the following convex optimization problem:

(9) $$\begin{array}{c} Min\\ w,b,{\text{ξ}}_{i\;},{{\text{ξ}}_{i\;}}^{*},\text{ε}\; \end{array}\;\frac{1}{2}\left|w^{T}w\right|+C(\text{νε}+\frac{1}{l}\sum_{i=1}^{i=l}\left({\text{ξ}}_{i\;}+{\text{ξ}}_{i}^{*}\;\right))$$

(10) $$Subject\;to\;\left\{\begin{array}{c} \begin{array}{c} (w^{T}\varphi \left(x_{i}\right)+b)-y_{i}\leq \varepsilon +{\text{ξ}}_{i}\\ y_{i}-(w^{T}\varphi \left(x_{i}\right)+b)\leq \varepsilon +{\text{ξ}}_{i}^{*} \end{array}\\ {\text{ξ}}_{i\;},{{\text{ξ}}_{i}}^{*}\geq 0,\;i=1,2,\ldots ,\;l,\;\;\varepsilon \geq 0 \end{array}\right\}$$

${\text{ξ}}_{i}$ and ${\text{ξ}}_{i}^{*}$ specify the upper and lower training errors subjected to the error tolerance, ε, while *C* is a positive constant which determines the trade-off between the flatness and the amount up to which deviations larger than ε are tolerated [23,25]. In this study, the LIBSVM, a Matlab library for SVM [26] is used to generate the proposed features based regression model for the SVR algorithm. The linear function is employed as a SVR mapping function for parameter C is 14.49 and *v* is −1.89 for both SBP and DBP models. These two parameters were selected with dynamic range from −20 to 20 and −20 to 0 for C and *v* respectively. The parameter b was 0.9865 and 0.6554 for SBP and DBP models respectively.

### 2.6. Evaluation of Results

In the present study, two standard protocols commonly used for the evaluation of the accuracy of blood pressure estimation, *i.e.*, the British Hypertension Association (BHS) and the American Association for the Advancement of Medical Instrumentation (AAMI) were applied. BHS evaluates the performance of the blood pressure estimation based on the cumulative percentage of readings which fall within absolute differences of 5, 10 and 15 mmHg from the mercury standard. The mercury standard refers to the SBP and DBP values obtained by a trained person using the auscultatory method (*i.e.*, using a stethoscope to listen to the Korotkoff sounds and a mercury sphygmomanometer to measure the pressure level in the cuff). To fulfil the BHS protocol, the tested device must achieve at least grade B, *i.e.*, 50% of readings falling within 5 mmHg, 75% within 10 mmHg and 90% within 15 mmHg of the readings obtained from the gold standard method, as illustrated in Table 2.

**Table 2:** Grading criteria according to the British Hypertension Society (BHS) protocol. Grades are derived based on the cumulative percentages of readings which fall within absolute differences of 5, 10 and 15 mmHg from the mercury standard. To achieve a particular grade, all three percentages must be equal to or greater than the tabulated values [27].

Grade	≤mmHg	≤10 mmHg	≤15 mmHg
CumUlative percentage of reading (%)			
A	60	85	95
B	50	75	90
C	40	65	85
D	Worse than C		

**Table 3:** Upper limit on the standard deviation of paired differences for given values of the mean of the paired differences (adapted from [28]).

Mean Difference	Standard Deviation
0	6.95 or less
±0.5	6.93 or less
±1.0	6.87 or less
±1.5	6.78 or less
±2.0	6.65 or less
±2.5	6.47 or less
±3.0	6.25 or less
±3.5	5.97 or less
±4.0	5.64 or less
±4.5	5.24 or less
±5.0	4.81 or less

On the other hand, to satisfy the AAMI standard, the mean difference between the measurements obtained from the tested device and from the gold standard method should lie within ±5 mmHg [28]. The upper limit on the standard deviation (SD) depends on the level of the mean difference, as listed in Table 3 [28].

### 2.7. Analyses

Two analyses were performed. In the first, we attempted to determine the effect of noise detection (outlier removal) on SBP and DBP estimation errors. In the second, we sought to establish the SBP and DBP estimation performance using conventional MAA, MLR and SVR models. The performance of the individual features will be evaluated followed by identification of the best combination of features for the two different types of blood pressure estimation models. We performed a comprehensive study on the performance of all possible combinations of two features on the blood pressure estimation methods, resulting in a total of 55 combinations. To search for the best combination of more than two indices, we applied SFFS starting from the best combination of two features provided by the exhaustive search. Our results revealed that adding a third feature did not provide an improvement in the results. A four-fold cross validation was applied during the implementation of all the blood pressure estimation methods.

## 3. Results

### 3.1. Effect of Noise Detection (Outlier Removal) on Systolic and Diastolic Blood Pressure Estimation Errors

**Figure 6:**
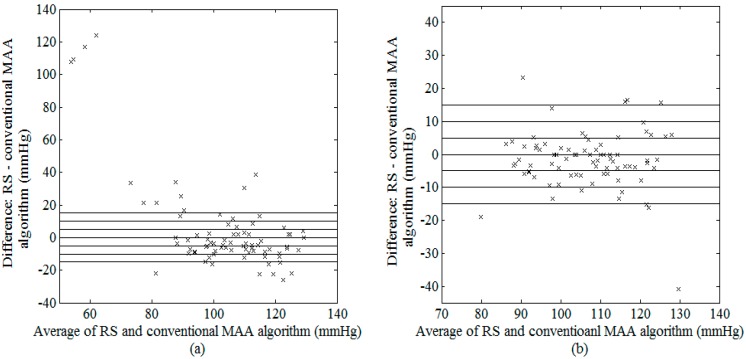
Bland–Altman plot of possible SBP between RS and conventional MAA algorithm (**a**) before and (**b**) after outlier removal.

**Figure 7:**
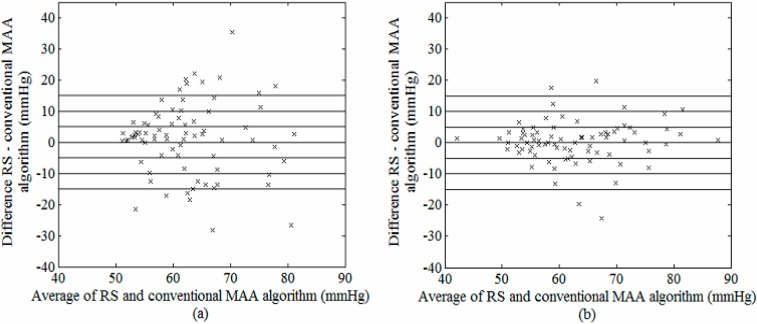
Bland–Altman plot of possible DBP between RS and conventional MAA algorithm (**a**) before and (**b**) after outlier removal.

Figure 6 and Figure 7 are the Bland-Altman plots demonstrating the performance of estimated SBP and DBP using the conventional MAA algorithm, with and without using the outlier removal algorithm before the OWE curve fitting process. On the other hand, cumulative percentage of blood pressure readings which fall within absolute differences of 5, 10 and 15 mmHg from RS (required for evaluation using the BHS standard) as well as mean ± SD difference between RS and conventional MAA algorithm (required for evaluation using the AAMI standard) were presented in Table 4. Based on the Bland–Altman plots for SBP (illustrated in Figure 6), the errors between the estimated pressure and the RS were large without outlier removal (up to 125 mmHg at low SBP), and substantially reduced upon elimination of the outlier points, with most data points lying within ±20 mmHg errors from the RS. Similar observations were found for the DBP (Figure 7). As shown in Table 4, the outlier removal method proposed in this study significantly improved the accuracy of the estimated pressures, with an improvement in BHS grades from D to B and A for SBP and DBP respectively. With regards to the AAMI standard, although a significant improvement was found in both mean and SD difference for SBP after outlier removal, the conventional MAA method failed to satisfy the AAMI standard (with a mean ± SD of −1.6 ± 8.6 mmHg, refer to Table 4).

In terms of DBP, the mean ± SD difference improved from 0 ± 14.2 mmHg to 0.3 ± 6.7 mmHg upon outlier removal, which satisfied the passing criteria for the AAMI standard.

**Table 4:** Cumulative percentage of readings which fall within absolute differences of 5, 10 and 15 mmHg from RS using the conventional MAA algorithm with the respective BHS grades, as well as mean ± SD and mean ± SD difference between RS and conventional MAA algorithm for blood pressure estimation before and after outlier removal.

	Grade	Cumulative Percentage of Reading (%)			Mean ± SD (mmHg)	Mean ± SD of Differences (mmHg)
	Absolute Difference: RS–MAA	≤5	≤10	≤15		
Before outlier removal						
SBP	D	30	61	74	101 ± 29	4.5 ± 28.6
DBP	D	43	61	79	63 ± 12	0.0 ± 14.2
After outlier removal						
SBP	B	55	84	90	107 ± 13	−1.6 ± 8.6
DBP	A	70	89	95	62 ± 9	0.3 ± 6.7

SBP, systolic blood pressure (range: 70–133 mmHg); DBP, diastolic blood pressure (range: 42–88 mmHg).

### 3.2. Systolic and Diastolic Blood Pressure Estimation Performance Using Conventional MAA, MLR and SVR Models

Table 5 and Table 6 showed the performance of each of the ten features extracted from the OWE in SBP and DBP estimation using the MLR and SVR models respectively. With regards to SBP, most features achieved a Grade B performance with both models according to the BHS standard, except for Area_3_ which obtained a Grade C performance using the MLR model. Individually, MAP as well as Ratio_2_ and Ratio_3_ derived based on the morphology of the OWE outperformed other features using both MLR and SVR models, as they provided lower mean and SD of differences between RS and estimated SBP values. In terms of DBP, most features achieved a Grade A performance according to the BHS protocol, except for Dur_2_ (Grade B using both MLR and SVR models), Dur_1_ and Ratio_3_ (both achieving Grade B with the SVR model). Based on the AAMI standard, comparable performances were observed among all ten features using both MLR and SVM models, with most features passing the AAMI standard marginally. Using the SFFS approach, we identified Ratio_2_ and Area_3_ to be the best combination of two features.

**Table 5:** Comparison among features extracted from the OWE envelope in blood pressure estimation performance using the MLR model.

Feature		Grade	Cumulative Percentage of Reading (%)			Mean ± SD (mmHg)	Mean ± SD of Differences (mmHg)
		Absolute Difference: RS–MLR	≤5	≤10	≤15		
Amp_1_	SBP	B	53	84	93	105±17	−1.2 ± 14.2
	DBP	A	70	92	95	63 ± 9	0.5 ± 6.1
Dur_1_	SBP	B	58	86	95	105 ± 13	−0.7 ± 10.6
	DBP	A	69	89	95	63 ± 10	0.6 ± 6.6
Dur_2_	SBP	B	54	79	91	105 ± 16	−1.1 ± 14.3
	DBP	B	63	87	94	63 ± 9	0.4 ± 6.8
Area_1_	SBP	B	50	84	93	105 ± 17	−1.3 ± 14
	DBP	A	70	89	95	63 ± 9	0.2 ± 6.2
Area_2_	SBP	B	61	86	94	105 ± 15	−0.4 ± 10.2
	DBP	A	71	92	95	63 ± 9	0.4 ± 6.2
Area_3_	SBP	C	50	79	86	104 ± 18	−1.9 ± 16.9
	DBP	A	73	89	95	63 ± 9	0.1 ± 6.3
Ratio_1_	SBP	B	55	80	93	106 ± 10	0.1 ± 8.0
	DBP	A	73	89	95	63 ± 9	0.3 ± 6.5
Ratio_2_	SBP	B	55	85	96	106 ± 10	0.5 ± 7.0
	DBP	A	68	89	96	63 ± 9	0.1 ± 6.6
Ratio_3_	SBP	B	55	85	96	106 ± 10	0.5 ± 7.0
	DBP	A	68	89	96	63 ± 9	0.1 ± 6.6
MAP	SBP	B	55	89	98	106 ± 10	0.3 ± 6.6
	DBP	A	71	89	95	63 ± 9	0.1 ± 6.6

SBP, systolic blood pressure (range: 70–133 mmHg); DBP, diastolic blood pressure (range: 42–88 mmHg).

As compared to the conventional MAA method using a fixed characteristic ratio (Table 7), the variable characteristic ratio method using both MLR and SVR models applied on the best combination of features significantly reduced the mean and SD of differences between the estimated SBP and that obtained from RS.

**Table 6:** Comparison among features extracted from the OWE envelope in blood pressure estimation performance using the SVR model.

Feature		Grade	Cumulative Percentage of Reading (%)			Mean ± SD (mmHg)	Mean ± SD of Differences (mmHg)
		Absolute Difference: RS–SVR	≤5	≤10	≤15		
Amp_1_	SBP	B	60	86	94	105 ± 18	−1.2 ± 15.4
	DBP	A	70	93	95	63 ± 9	0.4 ± 6.4
Dur_1_	SBP	B	60	88	94	104 ± 13	−1.7 ± 9.7
	DBP	B	65	89	94	64 ± 9	0.8 ± 6.4
Dur_2_	SBP	B	55	83	91	104 ± 15	−1.8 ± 13
	DBP	B	69	89	94	63 ± 9	0.7 ± 6.6
Area_1_	SBP	B	58	87	93	104 ± 16	−1.5 ± 15
	DBP	A	70	90	95	63 ± 9	0.2 ± 6.2
Area_2_	SBP	B	64	89	94	106 ± 14	−0.1 ± 9
	DBP	A	69	89	96	63 ± 9	−0.2 ± 6.4
Area_3_	SBP	B	53	81	91	104 ± 16	−1.5 ± 14
	DBP	A	70	91	95	63 ± 9	0.6 ± 6.5
Ratio_1_	SBP	B	58	84	94	106 ± 10	0.3 ± 7.6
	DBP	A	68	89	95	64 ± 9	1.0 ± 6.6
Ratio_2_	SBP	B	59	89	98	107 ± 10	1.0 ± 6.3
	DBP	A	70	90	95	63 ± 9	0.6 ± 6.6
Ratio_3_	SBP	B	58	88	98	107 ± 11	1.1 ± 6.4
	DBP	B	58	85	95	64 ± 9	1.4 ± 7.2
MAP	SBP	B	58	85	95	106 ± 10	0.2 ± 6.8
	DBP	A	70	89	95	63 ± 9	0.5 ± 6.7

SBP, systolic blood pressure (range: 70–133 mmHg); DBP, diastolic blood pressure (range: 42–88 mmHg).

**Table 7:** Comparison among conventional MAA method, MLR and SVR models in blood pressure (SBP and DBP) estimation performance using the best combination of features.

	Grade	Cumulative Percentage of Reading (%)			Mean ± SD (mmHg)	Mean ± SD of Differences (mmHg)
	Absolute Difference with RS	≤5	≤10	≤15		
Conventional MAA method (using fixed characteristic ratio)						
SBP	B	55	84	90	107 ± 13	−1.6 ± 8.6
DBP	A	70	89	95	62 ± 9	0.3 ± 6.7
MLR model (using the best combination of features)						
SBP	A	63	91	98	106 ± 11	−0.3 ± 5.8
DBP	A	71	89	95	63 ± 9	−0.2 ± 6.4
SVR model (using the best combination of features)						
SBP	A	66	94	98	107 ± 12	−0.6 ± 5.4
DBP	A	68	90	95	62 ± 9	0.4 ± 6.3
Method comparison (MLR- SVR)						
SBP	A	98	100	100		−0.3 ± 1.6
DBP	A	100	100	100		0.6 ± 1.0

SBP, systolic blood pressure (range: 70–133 mmHg); DBP, diastolic blood pressure (range: 42–88 mmHg).

Meanwhile, only a slight reduction in SD was observed for DBP. Based on the BHS standard, both MLR and SVR models, as well as the conventional MAA method could achieve a Grade A performance for SBP and DBP estimation. Generally, comparable performance was obtained for both MLR and SVR models, with up to 98% (95%) of data lying within ±15 mmHg from RS for SBP (DBP) estimation. In addition, both models satisfied the performance criteria set by the AAMI standard, with SVR model achieving a slightly lower SD of difference with RS but at a slightly higher mean difference value.

**Figure 8:**
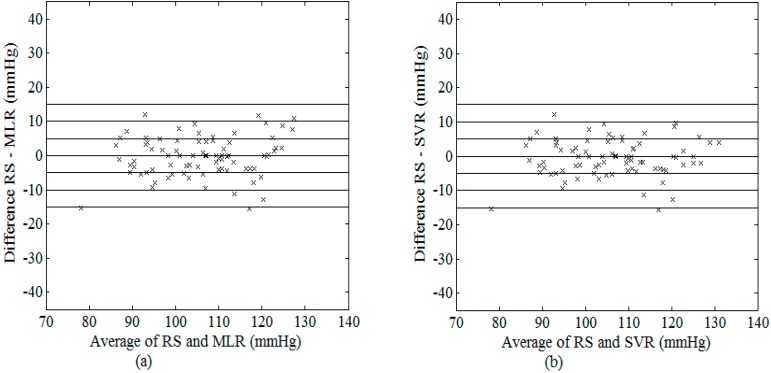
Bland–Altman plot of possible SBP between RS and (**a**) MLR model; (**b**) SVR model using the best combination of features.

**Figure 9:**
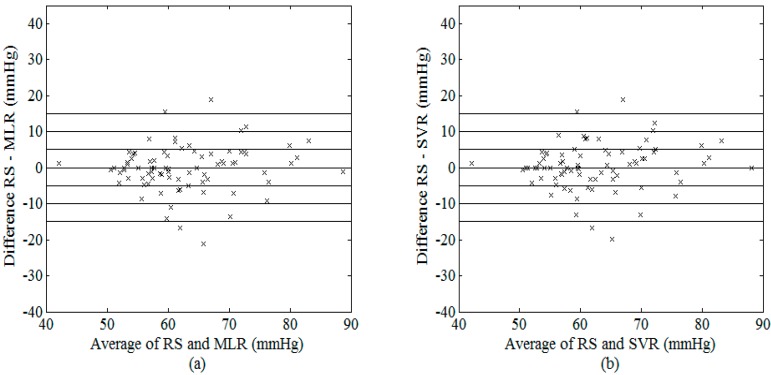
Bland–Altman plot of possible DBP between RS and (**a**) MLR model; (**b**) SVR model using the best combination of features.

**Figure 10:**
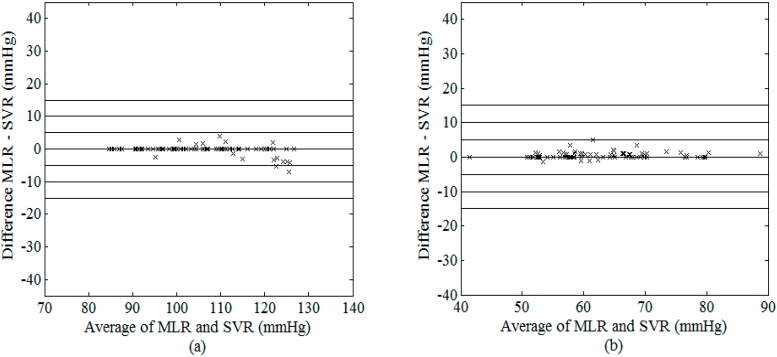
Bland–Altman plot of possible (**a**) SBP and (**b**) DBP between MLR and SVR models using the best combination of features.

As illustrated in Figure 8 and Figure 9, their estimated values for SBP (DBP) data used in the present study were very similar. Figure 10 shows the difference of SBP and DBP estimated between MLR and SVR, all the values lied within the range of ±5 mmHg (with the exceptions of only 2 values for SBP). At higher SBP values, SVR model appeared to provide larger values as compared to that estimated using the MLR model while an opposite trend was observed in the middle range of SBP.

## 4. Discussion

Accurate oscillometric blood pressure estimation in an unsupervised environment is challenging in the presence of interference, notably movement artifact which interrupts the air flow in the deflating cuff. While several studies have attempted to detect noise in the blood pressure signals using additional sensing devices such as acceleration and capacitive sensors [29], as well as morphological comparison with good-quality reference pulses [30], none of these studies have investigated the effect of the detected noise on the extraction of accurate blood pressure values from the contaminated signals. In the present study, we integrated an artifact removal block (Figure 1) in our SBP and DBP estimation algorithm which was based solely on the oscillometric signal without using additional sensors or reference signals. Our results demonstrated that the mean and standard deviation of the blood pressure estimation errors between the MAA algorithm and the RS substantially decreased upon artifact removal (Figure 6 and Figure 7, Table 4), which strongly advocates the importance of the artifact removal component proposed in the present study. Furthermore, the MAA algorithm has been well recognized to be susceptible to additive noise as it is derived based on the amplitude of the pulse [31]. The spline interpolation method, commonly used to smooth the envelope of the OMW for eliminating the erroneous peak values generated by artifact, was shown in this study to be ineffective in reducing the interference caused by movement artifact [32].

We further demonstrated from our analysis results (Table 5 and Table 6) that the usage of variable characteristic ratio derived based on several features extracted from the OWE improved the blood pressure estimation accuracy over the conventional MAA method using fixed characteristic ratios (SBP: mean ± SD = −1.6 ± 8.6 mmHg; DBP: mean ± SD = 0.3 ± 6.7 mmHg). Due to the large uncertainties in the characteristic ratios reported in the literature [7,12,33], we used averages of the SBPR and DBPR ratios derived from our reference SBP and DBP measurements based on the expert readings. When evaluated individually (Table 5 and Table 6), the MAP feature proposed by Lee *et al.* [15] as well as the two newly proposed features in the present study, *i.e.*, Ratio_2_ and Ratio_3_, outperformed other features in providing accurate SBP estimates (MAP: mean ± SD = 0.3 ± 6.6 mmHg for MLR and 0.2 ± 6.8 mmHg for SVR; Ratio_2_: mean ± SD = 0.5 ± 7.0 mmHg for MLR and 1.0 ± 6.3 mmHg for SVR; Ratio_3_: mean ± SD = 0.5±7.0 mmHg for MLR and 1.1 ± 6.4 mmHg for SVR). The Ratio_2_ and Ratio_3_ features were derived based on the morphology of the OWE, which has been reported to reflect the stiffness characteristics of the vessel [13]. Consistent with previously published findings, the degree of arterial stiffness and thus pulse pressure as well as the shape of the OWE has the largest influence on the SBP and DBP errors determined based on the conventional MAA method [12], leading to errors as high as 15%–20% [34] or 58 mmHg [12]. To the contrary, the Ratio_1_ feature proposed by Lee *et al.* [15], which also described the shape of the OWE, was found to be inferior in our study as compared to Ratio_2_ and Ratio_3_. The main difference between these features were that while Ratio_1_ was derived based on length of the oscillometric waveform, Ratio_2_ and Ratio_3_ described asymmetry in the waveform based on area of the OWE, thus took into consideration both amplitude and length of the waveform. Compared to length, measurements based on area of the OWE, e.g., Ratio_2_ and Ratio_3_ are more robust to noise interference as well as errors associated with difficulties in determining the starting and ending points of the cuff pressure oscillations. The Dur_2_, Amp_1_ and Area_1_ features proposed by Lee *et al.* [15] demonstrated poor performance in SBP estimates individually when applied on data set used in the present study.

Using the SFFS approach, we were able to achieve a significant reduction in the mean and standard deviation of differences between the estimated SBP values and the RS (MLR: mean ± SD = −0.3 ± 5.8 mmHg; SVR: mean ± SD = −0.6 ± 5.4 mmHg) with only two features, *i.e.*, Ratio_2_ and Area_3_ (Table 7), as compared to the conventional MAA method (mean ± SD = −1.6 ± 8.6 mmHg). To the contrary, negligible improvement was achieved for DBP estimation. Our results were comparable with that reported by Lee *et al.* [15], which utilized three features, *i.e.*, Area_1_, Ratio_1_ and MAP selected based on *t*-test evaluation on their clean dataset. While *t*-test evaluates the significance of features independently (filter-based method), the SFFS method takes into account interaction among features (wrapper-based method). The advantages of wrapper-based methods include taking into account feature dependencies [35], and they typically perform better in prediction accuracy when compared with filter-based methods [35].

Despite its simplicity, our results showed that the MLR model was able to achieve comparable performance with that obtained from the SVR model (Table 7, Figure 8, Figure 9 and Figure 10), which requires optimization of the model parameters through repeated training. The MLR model was able to estimate the best fitting surface of a suitable function that relates the independent and dependent variables [36]. On the other hand, Gaussian mixture regression [15] as well as Bayesian model [7], applied on a combination of five features, have also been recently proposed by Lee *et al.* [16] and evaluated on experimental data acquired from 85 healthy subjects. As compared to these methods, our MLR and SVR models do not need careful formulation of prior distributions of the data. In addition, the same research group has also presented a feature-based neural network approach for the estimation of blood pressure [16], which used features extracted from the OWE (consisting of the amplitudes, spreads, and centres of the modelled Gaussian functions) as inputs to the neural network. Although the proposed approach was shown to achieve lower values of mean and standard deviation of error in the estimations (SBP: mean ± SD = 6.76 ± 8.89 mmHg; DBP: mean ± SD = 5.98 ± 7.90 mmHg) as compared to the conventional MAA method, their results did not meet the AAMI standard. This was probably because their oscillometric measurements were taken at different time points with that acquired by the nurse, which served as RS. As suggested by Soueidan *et al.* [10], natural blood pressure variability often exceeds the maximum allowable error set by the AAMI standard (*i.e.*, ±5 mmHg), thus it is advisable to acquire simultaneous recordings of both oscillometric signal and RS for accurate comparison, as that performed in the present study.

Using a different approach based on a Fourier series representation of the oscillometric waveform, Barbe *et al.* [37] introduced a Hammerstein-Windkessel model which captures the low frequency oscillations of the cardiovascular system. The systolic and diastolic pressures were derived from the mean arterial pressure using an intuitive estimator α, which was calculated based on the envelope of the modeled oscillometric waveform. The α parameter, which reflects the symmetry of the oscillometric waveform, is similar to one of the best performing feature in the present study, *i.e.*, Ratio_3_. In a more recent study, they [17] further extended their work to include a statistical learning technique based on ordinal logistic regression for the calibration of oscillometric blood pressure monitors. By applying a linear regression to map the shape of the oscillometric signal to the blood pressure to avoid complex nonlinear models, the method could only estimate the correct blood pressure range but not the specific value of the blood pressure.

One limitation of the present work is that the experimental measurements were obtained from healthy subjects and the measurements were conducted in a laboratory environment. Ongoing studies are carried out to record signals directly from unsupervised environments in different cohorts of subjects to assess the robustness of our algorithms in a wider range of subjects.

## 5. Conclusions

In this study, we proposed a novel approach in estimating SBP and DBP using variable characteristic ratios derived from features extracted from the OWE, on data corrupted with movement artifact. An automatic algorithm based on changes in the oscillometric pulses relative to their respective neighbour pulses was proposed to remove outlier points before the curve fitting process. Substantial reduction in the mean and standard deviation of the blood pressure estimation errors between the MAA algorithm and the RS were obtained upon artifact removal. Comparing all ten features extracted from the OWE, the MAP feature as well as the two newly proposed features, *i.e.*, Ratio_2_ and Ratio_3_, showed superior performance in providing accurate SBP estimates. Using SFFS, we were able to achieve a significant reduction in the mean and standard deviation of differences between the estimated SBP values and the RS (MLR: mean ± SD = −0.3 ± 5.8 mmHg; SVR and −0.6 ± 5.4 mmHg) with only two features, *i.e.*, Ratio_2_ and Area_3_, as compared to the conventional MAA method (mean ± SD = −1.6 ± 8.6 mmHg). To the contrary, negligible improvement was achieved for DBP estimation. Comparing both MLR and SVR models, our results showed that the MLR model was able to achieve comparable performance with that obtained from the SVR model despite its simplicity.
